# Gillespie syndrome: An atypical form and review of the literature

**DOI:** 10.1016/j.amsu.2022.103244

**Published:** 2022-01-08

**Authors:** O. Nabih, H. Hamdani, L. ELMaaloum, B. Allali, A. ELkettani

**Affiliations:** Pediatric Ophthalmology Department, Ho^pital 20, Aou^t 1953, Casablanca, Morocco

**Keywords:** Gillespie syndrome -partial aniridia, Scalloping iris, Vermian hypoplasia

## Abstract

**Introduction:**

Gillespie syndrome (GS) is a rare genetic disorder that combines ocular and cerebral defects.

It was first described in 1965, by Frederick D. Gillespie. He reported a triad of congenital aniridia, cerebellar ataxia and mental retardation in a 22-year-old woman and her 19-year-old brother. Its etiology is still unknown.

To date, less than 30 patients have been reported in the literature.

**Observation:**

We report the case of a 2 years old child, born of a consanguineous marriage. At the age of 8 months, the parents consulted for a delay in psychomotor acquisition for which the MRI performed showed a vermian hypoplasia. It was only at the age of 2 years, following a contusive trauma of the left eye that a partial aniridia was objectified on both eyes associated with a lens coloboma on the left eye. In view of these clinico-radiological data, the diagnosis of Gillespie syndrome was retained.

**Discussion:**

Gillespie syndrome is a genetic disease. It combines ocular and neurological abnormalities. It was first described in 1965 by Gillespie. The ocular manifestations of Gillespie syndrome mainly concern the iris. Aniridia is always present with, in most cases, a scalloped appearance of the pupillary margin. It can be accompanied with additional ocular findings such as foveal, optic nerve hypoplasia, retinal hypopigmentation, and/or pigmentary macular changes leading to reduced visual acuity.

In addition to ocular abnormalities, the Gillespie syndrome. (GS) includes neurological deficiencies, particularly axial hypotonia, lack of coordination, dysarthria and static and kinetic ataxia.

**Conclusion:**

The diagnosis of Gillespie Syndrome should be evoked in any hypotonic child presenting with bilateral but partial aniridia. Prognosis depends on the proper management and anticipation of ocular and mental symptoms and disabilities.

## Highlights

• Gillespie syndrome (GS) is a rare genetic disorder that combines ocular and cerebral defects.
• Since Gillespie's publication, few additional cases have been reported in the literature.
• We describe the clinical feature of ocular involvement which is clinically distinct from other forms of aniridia and may be pathognomonic of Gillespie syndrome.

## 1 Introduction

Gillespie syndrome (GS) is a rare genetic disorder that combines ocular and cerebral defects. In 1965, Frederick D. Gillespie described a triad of congenital aniridia, cerebellar ataxia and mental retardation in a 22-year-old woman and her 19-year-old brother.

Since Gillespie's publication, few additional cases have been reported in the literature. We add a new case to the list, emphasising the characteristic of the ocular involvement, which is clinically distinct from other forms of aniridia and may be pathognomonic of Gillespie syndrome.

This study has been reported in accordance with the SCARE criteria [1].

## 2 Observation

**Fig. 1:**
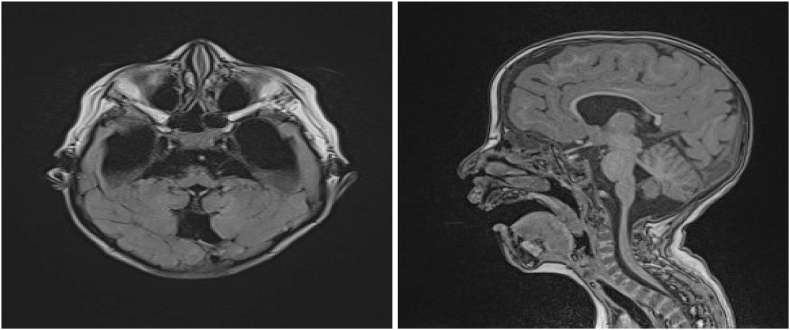
Brain MRI with axial FLAIR and sagittal T1 sequence showing partial hypoplasia of the inferior vermis with communication of the 4th ventricle and the large cistern and ventricular dilatation without other associated malformations.

We report the case of a 2-year-old child of first-degree consanguineous parents, born at term at 38 AW by vaginal birth, with a low birth weight of 2.2 kg. He presented during delivery a neonatal respiratory distress due to inhalation of amniotic fluid, for which he was admitted to a neonatal intensive care unit with a good clinical evolution. At the age of 8 months, the parents consulted for delayed psychomotor acquisitions for which a brain scan was performed showing vermian hypoplasia [Fig. 1].

**Fig. 2:**
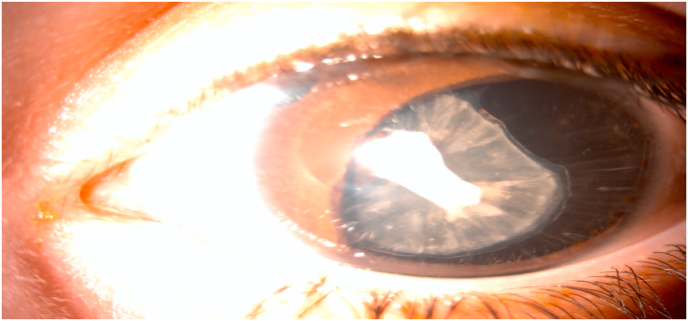
Slit lamp image showing partial temporal aniridia with opalescent lens and superior temporal lens coloboma.

**Fig. 3:**
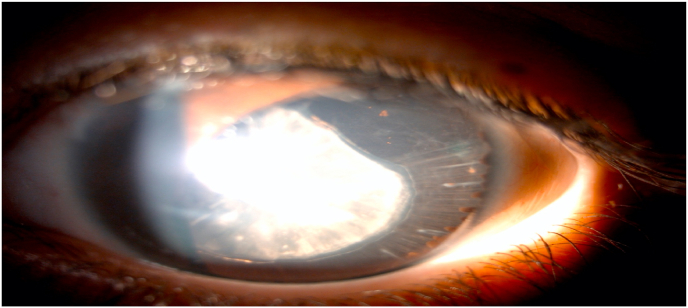
Slit lamp image showing temporal iridodysgenesis with a scalloped iris stump.

At the age of two, the parents consulted at the ophthalmological emergency room after a contusive ocular trauma of the left eye with the appearance of leucocoria and increasingly evident photophobia. At this age, the child had not yet acquired either sitting or speaking. The initial ophthalmological examination showed good eye tracking without nystagmus. Ophthalmological examination with sedation revealed clear corneas, normal corneal diameter at 11mm, and deep anterior chamber. There was a distinctive partial bilateral aniridia. The lens was opalescent in the left eye, with a superior temporal lens coloboma and a clear lens on the right [Fig. 2, Fig. 3].

Intraocular pressure (IOP) was less than 6 mmHg in both eyes. Gonioscopy showed iridodysgenesis with residual iris stump, anterior inferior appositions and no identifiable synechiae.

The fundus showed a normal coloured papilla with regular contours, a good foveolar reflection with perimacular hypopigmentation, vessels of normal size and diameter without arteriovenous crossover. An ophthalmological paraclinical test involving evoked potentials and an electroretinogram came back normal. Abdominal, pelvic and cardiac ultrasound were normal. The urinary metabolic work-up and serologies for infectious fetopathy were normal. A genetic study was not performed due to lack of means.

## 3 Discussion

Gillespie syndrome is a genetic disease. It combines ocular and neurological abnormalities. It was first described in 1965 by Gillespie [2].

Including our patient, a total of 27 cases of Gillespie syndrome have been described to date. Nine cases are sporadic and 18 cases belong to 10 different families [3].

Genetically, no mutations have been identified [4]. However, Dollfus et al. [5] found a de novo translocation t(X; 11) (p22.32; p12) in a patient with this syndrome, without any correlation with the involvement of the PAX6 gene, which is located in the 11p13 region and expressed very early in the brain and in a large part of the embryonic structures of the eye.

Congenital aniridia, unlike gillespie syndrome, is autosomal dominant due to mutation of the PAX6 gene [6]. These are therefore two genetically different conditions. This suggests that gillespie syndrome is genetically heterogeneous [7].

Studies have shown that the association of cerebellar damage with congenital aniridia argues for a failure of neuroectodermal and mesodermal development during embryonic life [8].

The usual clinical presentation is the discovery of dilated pupils in a hypotonic infant. The pupils appear mydriatic and unresponsive to light and accommodation. There is no response to the application of myotic or mydriatic drops. Loss of the iris sphincter responsible for pupillary dilation and iris aplasia are often present. Partial aniridia is variable from patient to patient. The pupillary edge of the iris usually has a “scalloped” edge on slit-lamp examination, extending over the anterior surface of the lens at regular intervals [[7], [8], [9]].

Lechtenberg et al. [10] reported bilateral ptosis with intermittent right exotropia in their 18 month old patient.

Pupillary membrane residues are also frequently present. Generally, the cornea and lens are clear. The distinctive feature of our observation is that there was a probably post-traumatic cataract with a lens coloboma. The fundus is usually normal.

In contrast, patients with “classic” autosomal dominant or sporadic aniridia usually have poor vision and nystagmus. This is due to associated foveal hypoplasia. In sporadic aniridia, the iris may be totally absent or if any remains are present, they tend to be irregular and asymmetric. Over time, keratopathy develops, cataracts and lens subluxation may be present. Secondary glaucoma sometimes occurs and the fundus usually shows macular hypoplasia with or without optic nerve hypoplasia.

Children with Gillespie Syndrome should be monitored regularly because of the very high risk of glaucoma. Diagnosis and correction of any myopia is necessary.

In our case, the child had undergone cataract surgery with intracapsular lens extraction and optical correction with aphakic glasses.

In addition to ocular abnormalities, the Gillespie syndrome. (GS) includes neurological deficiencies, particularly axial hypotonia, lack of coordination, dysarthria and static and kinetic ataxia. This cerebellar ataxia becomes evident over the course of the disease [10,11]. Mental retardation is present in the majoritý of patients [11].

Radiologically, brain CT or better magnetic resonance imaging (MRI) shows cerebellar hypoplasia or atrophy prevailing on the vermis [7,10,12]. Other cerebral white matter and cerebellar abnormalities may exist [7].

In addition to iridocerebellar anomalies, a number of other osteoarticular, vascular and atrial malformations have been described in GS: C1-C2 vertebral fusion [12] or C2-C3 associated with odontoid hypoplasia [7]; pulmonary artery stenosis [2,12], and helix hypoplasia [2].

The diagnosis of GS was retained in our patient on the association of bilateral but partial aniridia, cerebellar ataxia, and delayed psychomotor acquisitions with hypoplasia of the cerebellar vermis on imaging. We eliminated the other differential diagnosis eventually Marinesco-Sjogren syndrome which associates aniridia with a congenital but not post-traumatic cataract, cerebellar ataxia and mental retardation.

## 4 Conclusion

The diagnosis of Gillespie Syndrome should be evoked in any hypotonic child presenting with bilateral but partial aniridia. Cerebellar signs and mental retardation are confirmed with age. The ocular signs are not pathognomonic of the disease, therefore the cytogenetic study is important.

## Ethical approval

This type of study does not require any ethical approval by our institution.

## Sources of funding

This study did not receive any sources of funding.

## Author contribution

O.Nabih: drafting the article, study concept, writing the article. H.Hamdani: acquisition of data. L.El maaloum: study design. B.Allali: revising the article. A. El kettani: final approval.

## Registration of research studies

1. Name of the registry:

2. Unique Identifying number or registration ID:

3. Hyperlink to your specific registration (must be publicly accessible and will be checked):

## Guarantor

O.NABIH.

## Provenance and peer review

Not commissioned, externally peer-reviewed.

## Declaration of competing interest

The authors declare no conflict of interest.
